# A Novel Homozygous Six Base Pair Deletion Found in the *NFATC2* Gene in a Patient with EBV-Associated Lymphoproliferation

**DOI:** 10.1007/s10875-024-01675-z

**Published:** 2024-03-01

**Authors:** Baran Erman, Sevgi Köstel Bal, Çiğdem Aydoğmuş, Gizem Zengin Ersoy, Kaan Boztug

**Affiliations:** 1 Institute of Child Health, Hacettepe University, Ankara, Türkiye; 2 Can Sucak Research Laboratory for Translational Immunology, Hacettepe University, Ankara, Türkiye; 3 St. Anna Children’s Cancer Research Institute, Vienna, Austria; 4 Ludwig Boltzmann Institute for Rare and Undiagnosed Diseases, Vienna, Austria; 5 Division of Pediatric Allergy and Immunology, Basaksehir Cam and Sakura City Hospital, University of Health Sciences, Istanbul, Turkey; 6 Department of Pediatric Hematology Oncology and Pediatric Bone Marrow Transplantation, Medical Park Bahçelievler Hospital, Altınbaş University, İstanbul, Turkey; 7 CeMM Research Center for Molecular Medicine of the Austrian Academy of Sciences, Vienna, Austria; 8 St. Anna Children’s Hospital, Vienna, Austria; 9 Department of Pediatrics and Adolescent Medicine, Medical University of Vienna, Vienna, Austria

**Keywords:** Combined immunodeficiency, *NFATC2*, EBV-associated lymphoproliferation

**To the Editor,**

The Nuclear Factors of Activated T cells (NFATs) family of transcription factors comprises 5 proteins (NFAT1-5) essential for T-cell activation [1]. NFAT1-4 proteins function on a calcium–calcineurin dependent signalling cascade which leads to nuclear localization of these transcription factors [2]. Activated calcineurin by downstream T-cell receptor (TCR) signals dephosphorylates NFATs, resulting in binding the interleukin-2 (IL-2) promoter region in the nucleus. The other specific roles of NFAT family members include follicular helper T-cell (Tfh) differentiation, regulation of immunological tolerance, and the immune metabolism and the control mechanism of T-cell anergy and exhaustion [2]. A novel type of syndromic inborn error of immunity has been described recently, caused by biallelic loss-of-function variants in the *NFATC2* gene encoding for NFAT1 [3]. Here we identified NFAT1 deficiency caused by a homozygous deletion in *NFATC2* gene in a patient with EBV (Epstein-Barr virus)-associated inborn error of immunity.

The index patient is a 12-year-old girl born to first-degree consanguineous parents. She was referred to immunology clinic due to possible recurrent chest infections, lung deterioration, chronic wet cough and failure to thrive at the age of 9. Her elder brother died of lymphoma when he was 5 years old, otherwise family history was unremarkable. Physical examination revealed bilateral crackles on auscultation and generalized lymphadenopathies. In the laboratory findings, severe hypogammaglobulinemia (IgG: 390 mg/dl, IgA: < 10 mg/dl, IgM: 99 mg/dl) together with low vaccine (Anti-HBs: -) and isohemagglutinin responses (Anti-A: ½, Anti-B: ½) were detected. Absolute lymphocyte counts and the main subset distribution of T and B cells were normal in the initial evaluation (Supplementary Table 1). EBV PCR was positive with a high copy number of 1.0 × 10^6^ /ml, prompting a possible diagnosis of EBV-related combined immunodeficiency. Any bacterial, fungal or viral infection agent in sputum or endotracheal aspirate culture were not detected. IVIG (intravenous immunoglobulin) replacement therapy together with trimethoprim-sulfamethoxazole prophylaxis was initiated. Lymph node biopsy showed EBV-related lymphoproliferative disease with EBER antigen positivity and non-suggestive findings of lymphoma. We concluded that EBV-related lymphoproliferation might contribute the development of lung deterioration in the patient. Following 6 months she received acyclovir therapy, however, it was not sufficient to control the EBV copy numbers. Due to lymphadenopathy, lymphoproliferation and malignity risk, four courses rituximab (375 mg/m2) were given. Although the EBV copy numbers were decreased, it was not negative and additional 4 courses rituximab were continued. After the 6. course, EBV infection was eradicated. Due to immunodeficiency, chronic EBV viremia and uncontrolled lymphoproliferation, the patient was planned for allogeneic hematopoietic stem cell transplantation (HSCT) from her healthy HLA-identical sibling. Following a treosulfan/fludarabine conditioning regimen, peripheral stem cells were infused at a dose of 5.7 × 10^6^ CD34 + cells/kg body weight. Post-transplantation thrombocyte, lymphocyte and neutrophil engraftment occurred on days + 10, + 11, and + 12 respectively. Acute skin and gastrointestinal graft versus host disease (GvHD) manifested on posttransplant 2 months which developed into chronic skin and lung GvHD. She was treated with multiple immunosuppressive agents including corticosteroids, ruxolitinib, cyclosporin A, MMF, imatinib as well as mesenchymal stem cells and extracorporeal photopheresis. In the 3rd year after HSCT she retained full donor chimerism, exhibited normal lymphocyte subsets and immunoglobulin levels, and EBV infection did not occur again. However, she suffered from chronic skin and lung GvHD, necessitating a lung transplantation. While she was waiting for a donor, she succumbed to a respiratory failure following a severe Klebsiella sepsis (Fig. 1A).

**Fig. 1:**
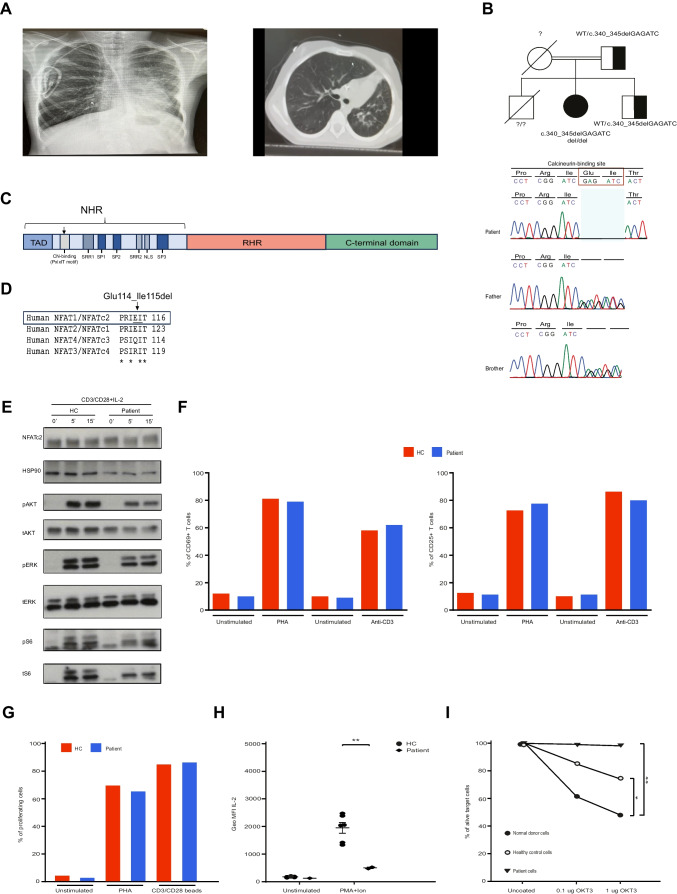
**A** Chest X-Ray and CT scan images of the patient indicating volume loss and interstitial infiltration in the left lung. **B** The pedigree of the family and the Sanger chromatograms indicated the possible causative deletion in patient and the heterozygous family members. **C** Domain architecture of the NFAT1 protein. The p.Glu114_Ile115del variant indicated by the black arrow deletes two amino acids of the conserved PxIxIT motif within the CN-binding domain. **D** Conservation of the PxIxIT motif across human NFAT1-4. The deleted residues in the patient are underlined. **E** Expressions of NFAT1 and proteins involved in downstream of TCR signaling by Western blot. **F** Comparable levels of CD69 and CD25 expressions on T lymphocytes on day 1 and 2 after TCR stimulation via anti-CD3 or PHA. **G** T lymphocyte proliferation in the patient, compared to healthy control. T cells were stimulated with PHA and CD3/CD28 beads for 3 days and the % of proliferating cells were determined by CFSE labeling. **H** Intracellular IL-2 production in the patient and a healthy control. PBMCs were stimulated with PMA + Ion for 5 h. Results obtained from at least 3 independent experiments. **I** Cytotoxicity measurement by CD8 + T cell killing assay. % of alive target cells analysed after 4 h of OKT-3 stimulation. Two tailed student’s t-test was used for the comparisons between the data of the patient and control samples. WT: Wild type, CN: Calcineurin, NHR: NFAT homology region, NLS: Nuclear localization signal, RHR: Rel homology domain, SP: SPXX-repeat motif, SRR: Serine-rich region, TAD: Transactivation domain, HC: Healthy control, PHA: Phytohemagglutinin. CFSE: Carboxyfluorescein succinimidyl ester (* < 0.05, ** < 0.01)

Given the consanguinity, we performed whole exome sequencing to identify a potential genetic etiology of the disease. Under the assumption of autosomal recessive inheritance, sequencing analysis revealed a homozygous deletion resulting the lack of 2 amino acids in the *NFATC2* gene (NM_012340.4, c.340_345delGAGATC, p.Glu114_Ile115del). The variant was validated by capillary sequencing. The father and the healthy brother of the patient were heterozygous for the variant (Fig. 1B), but material from the mother was not available as she had passed away due to breast cancer. The SIFT and Provean prediction scores indicated the variant as damaging and deleterious, respectively and the CADD score was 21. Inherited deletion affects the conserved PxIxIT motif within the calcineurin-binding domain of NFAT1 (Fig. 1C and D).

Detailed lymphocyte subpopulation analysis during rituximab therapy (at the age of 12) revealed CD4 + CD45RA + and CD4 + CD45RO + naive and memory T cells, respectively, were almost equal (49.35% and 49.33%). There was a relative expansion of the CD4^+^CCR7^−^ effector T-cell subset. Both CD19 + and CD20 + B cells were almost not detectable likely due to rituximab treatment. The percentage and number of NK cells were also diminished (Supplementary Table 1). The expression of NFAT1 and proteins involved in downstream of TCR signalling were normal by western blot (Fig. 1E). To assess the functional responses, we studied activation and proliferation of T cells, which were comparable to healthy controls (Fig. 1F and G). IL-2 production following PMA/Ionomycin stimulation was significantly lower in patient lymphocytes in comparison with the healthy control (Fig. 1H). Cytotoxic killing function of patient’s lymphoblasts was impaired in a target cell killing assay (Fig. 1I).

Since NFAT1 protein has a pleiotropic role in different human cell types, NFAT1 deficiency may affect various pathways in immune cells. Recently, Sharma and colleagues identified complete NFAT1 deficiency in a patient with progressive joint contractures, osteochondromas, and B-cell malignancy [3], caused by a homozygous 4-nucleotide deletion leading a premature stop codon in the *NFATC2* gene. The immune phenotype of this patient was remarkable for accumulation of naive B cells, exhausted CD4 + T cells, reduced T follicular helper and CD8 + T cells, in addition to impaired cartilage cell growth and differentiation. In our patient, only CD4 + effector memory cells were increased and unlike the first identified patient, there is no soft tissue or cartilage abnormalities. The variant identified in our study is located in the calcineurin binding site and likely affects the translocation of the NFAT1 protein to the nucleus. Despite the fact that *NFATC2* knockout mice model and complete NFAT1 deficiency certified the impaired regulation of chondrogenesis [3, 4], there may be residual NFAT1 function in our patient. This may be the possible explanation for the absence of profound cartilage/joint issues in the patient. Future identification of additional patients harbouring missense variants in *NFATC2* will enable a more comprehensive understanding of genotype-phenotype correlations in this rare inborn error immunity.

The differentiation and effector functions of CD8 + T cells regulate by NFAT1 and NFAT2 [5] and the impaired cytotoxicity of CD8 + T cells with diminished IL-2 production have been previously shown in the studies by Klein-Hessling and our own data [6, 7]. We consider that the decreased killing activity is the possible cause of the EBV-associated lymphoproliferation in our patient. In contrast to cytotoxic T lymphocytes, NFAT1 was shown as a negative regulator of NK-cell function in mice [8]. However, progressive decrease of NK-cell numbers in the patient’s follow up might had an additional effect on uncontrolled EBV-associated proliferation. Although IL-2 production via PMA/Ionomycin stimulation was diminished in our patient’s T cells, the activation and proliferation were intact in CD3 + T lymphocytes. We think that these mechanisms could be compensated by NFAT2 [9] or an IL-2 and NFAT independent manner induced by CD28 stimulation [10]. In murine models, *NFATC2* knockout (KO) CD4 + CD252 ‘conventional’ T cells displayed increased proliferation after CD3 stimulation [11]. In the same study, generalized lymphadenopathy and B-cell malignancies were developed in *NFATC2* KO mice which may explain uncontrolled B cell lymphoproliferation in our patient. Unfortunately, we could not perform detailed functional tests for impaired B-cell development and differentiation due to progressive loss of B lymphocytes after rituximab treatment, and similarly, the allogenic HSCT prevent us from being able to characterize the T-cell compartment in further depth. Notably, although the phenotype of the patient in our study is suggestive that the NFATC2 lesion may be causative for the observed phenotype, the lack of additional primary patient material made it impossible to demonstrate causality of the NFATC2 for the observed clinical and immunological phenotype in a strict sense accordingly to outlined criteria by distinguished investigators in the field [12].

In conclusion, we may have identified an additional patient with an inborn error of immunity linked to a genetic defect in NFATC2. We speculated that, similar to other IEIs, NFAT1 deficiency may present with a spectrum of clinical phenotypes, since NFAT family proteins play different key roles in immune system and the types of the variants may affect the immune mechanisms in different ways cause diverse phenotypes. Hence, several NFAT family variants such as in *NFATC1*, *NFATC2* and *NFAT5* genes have been identified with different inborn errors of immunity [3, 7, 13]. Since only two patients have been identified with NFAT1 deficiency so far, more patients with longer follow up are needed to elucidate this rare inborn error of immunity. In addition, HSCT may be considered as a therapeutic strategy since successful immune reconstitution was established with the control of hypogammaglobulinemia and EBV infection in our patient.

### Supplementary Information

Below is the link to the electronic supplementary material.

Supplementary file1 (PDF 564 KB)

## Data Availability

There is no dataset generated during the study.
