# Supplementary material for: A Novel Homozygous Six Base Pair Deletion Found in the NFATC2 Gene in a Patient with EBV-Associated Lymphoproliferation
Source: J Clin Immunol. 2024 Mar 1;44(3):74. doi: 10.1007/s10875-024-01675-z (PMC10907409; doi:10.1007/s10875-024-01675-z)
Supplement: Supplementary file 1 — Supplementary file1 (PDF 564 KB) [file 10875_2024_1675_MOESM1_ESM.pdf]

A novel homozygous six base pair deletion found in the *NFATC2* gene in a patient with EBV-associated lymphoproliferation

Journal of Clinical Immunology

Baran Erman[1,2]*, Sevgi Köstel Bal[3,4]*, Çiğdem Aydoğmuş[5], Gizem Zengin Ersoy[6], Kaan Boztug[3,4,7,8,9]

[1] Institute of Child Health, Hacettepe University, Ankara, Türkiye

[2] Can Sucak Research Laboratory for Translational Immunology, Hacettepe University, Ankara, Türkiye

[3] St. Anna Children's Cancer Research Institute, Vienna, Austria

[4] Ludwig Boltzmann Institute for Rare and Undiagnosed Diseases, Vienna, Austria

[5] Division of Pediatric Allergy and Immunology, Basaksehir Cam and Sakura City Hospital, University of Health Sciences, Istanbul, Turkey

[6] Department of Pediatric Hematology Oncology and Pediatric Bone Marrow Transplantation, Medical Park Bahçelievler Hospital, Altınbaş University, İstanbul, Turkey

[7] CeMM Research Center for Molecular Medicine of the Austrian Academy of Sciences, Vienna, Austria.

[8] St. Anna Children's Hospital, Vienna, Austria

[9] Department of Pediatrics and Adolescent Medicine, Medical University of Vienna, Vienna, Austria

Correspondence:

Baran Erman, Beytepe Campus of Hacettepe University HUNITEK Building Floor 1 06800 Ankara Türkiye, +90 312 297 63 61, baranerman@gmail.com

Supplementary Methods

Study Participants

The blood samples of the patient and healthy control were obtained in accordance with the local Ethics Committee of the Hacettepe University. Informed consent forms were provided by the participants.

Mutation Analysis

Genomic DNA was extracted from the peripheral blood using DNA isolation kit (GeneAll). The NGS exome library was prepared using Nextera DNA Prep with Enrichment Kit (Illumina). 150-bp paired-end sequencing was performed using the Illumina NextSeq 550 platform. The mapping, variant calling and annotation were analyzed with SEQ Platform v8 (Genomize). To validate the identified *NFATc2* variant, Sanger sequencing was performed in accordance with the standard protocols [1]. Primers used for PCR amplification and Sanger sequencing were forward, 5' GCATACCCCGATGATGTCCTT 3' and reverse, 5' TTATTGGGCGAGACGCAGG 3'. The amplified DNA region was subjected to sequencing using ABI 3130xl Genetic Analyzer (Applied Biosystems).

The potential disease-causing homozygous variants from the filtered exome data were listed in Supplementary Table 2.

Flow Cytometry

Standard flow cytometric methods were used for staining of cell-surface proteins. Flow cytometric tests were performed on FACSCelesta (BD Biosciences). Data were analyzed with FACSDiva (BD Biosciences) and FlowJo software (BD Biosciences). Following antibodies used for staining: CD3-PE (OKT3), CD3-APC (OKT3), CD4-BV510 (SK3), CD8-BV421 (SK1), CD69-PE(FN50), CD45RO-BV421 (UCHL1), CD8-PERCP (BD, SK1), CD25-FITC (M-A251), CD45RA-APC (HI100), CD56-AF488 (B159), CD197-PE (G043H7), CD19-APCCy7 (SJ25C1), CD16-APC (B73.1), CD38-APC (HIT2), IgD-BV421 (IA6-2), CD27-APC (M-T271), IgM-FITC (G20-127), CD185-FITC (J252D4), CD31-PE (WM59).

Western Blotting

For Western blotting, PBMCs (peripheral blood mononuclear cells) from healthy control and patient were stimulated with CD3/CD28 Dynabeads (Thermo) and human recombinant IL-2 (Biolegend) for 5 or 15 minutes. Cell lysates were obtained using Xtractor Buffer (Takara). Proteins were quantified using DC Protein Assay (Bio-Rad) and 20 µg of total protein was loaded on 4-12% precast polyacrylamide gel (Bio-Rad). After proteins were transferred to PVDF membrane by Semi Trans-Blot System (Bio-Rad), the membrane was blocked with 3% BSA in TBST. Blots were incubated overnight with the following primary antibodies; anti-human NFAT1 (Thermo), HSP90 (Thermo), phospho AKT (Biolegend), AKT (Biolegend), phospho ERK (Biolegend), ERK (Biolegend), phospho S6 (Thermo) and S6 (Thermo). HRP-conjugated anti-rabbit IgG antibody (BioLegend) was used for secondary antibody incubation for 1 hour. Blots were observed by ChemiDoc Imaging System (Bio-Rad).

Cell culture, lymphocyte activation and proliferation assays

PBMCs were isolated from the patient and healthy controls using Ficoll-Paque (Capricorn Scientific). Cells were resuspended in RPMI-1640 containing 10% FCS, 10 mM HEPES (Sigma), 100 U/ml penicillin (Sigma) and 200 mM L-Glutamine (Sigma). For proliferation assays, cells were stimulated with CD3/CD28 Dynabeads (Thermo) or 5 µg/ml PHA (Phytohemagglutinin) (Sigma) for 3 days. Proliferation was measured by labeling the cells with 5 µM CFSE (BioLegend) according to manufacturer's instructions. For activation assays, cells were stimulated with anti-CD3 (Thermo) or 5 µg/ml PHA for 24 and 48 hours for CD69 and CD25 expressions on T cells, respectively.

IL-2 measurement

Intracellular IL-2 production was analyzed by stimulating 0.2×10^6 total PBMCs for 5 h with Phorbol 12-myristate 13-acetate (PMA, 0.2 mM) and Ionomycin (1 µg/mL) and adding Brefeldin A during the final 2.5 h of the stimulation. Cells were stained for T-cell surface marker, CD3 on ice for 30 mins. Subsequently, cells were fixed, permeabilized and stained with anti-IL-2.

Cytotoxicity assay

GFP-expressing P815 target cells were treated with aphidicolin to inhibit proliferation and pre-incubated either with or without OKT-3 (1 µg/ml). Different ratios of target cells and CD8+ T cells

were distributed in 96-well U-bottom low-cell-binding plates (Nunc). Following 4 hours of incubation, cells were stained with 7-AAD and residual alive target cells were evaluated.

Statistical analysis

Statistical analysis was performed by using Graphpad Prism software. Two tailed student's T-test was used for the comparisons between the data of the patient and healthy control.

Supplementary Table 1. Laboratory findings of the patient

**Complete blood cell counts** (At the age of 9)	Results	Reference values [2, 3]
Leukocytes (10^3 /µl)	10.1	3.7-11.1
Lymphocytes (10^3 /µl)	4.5	1.5-7.6
Neutrophils (10^3 /µl)	6.1	1.78-5.38
Platelets (10^3 /µl)	280	140-450
Monocytes (10^3 /µl)	0.5	0.3-0.82
**Lymphocyte subsets** (At the age of 9)	**%/count (10^3 /µl)**	
CD3	79/3.6	58-82/1.1-4.1
CD4	47/2.1	26-47/0.6-2.4
CD8	31/1.4	16-32/0.4-1.5
CD19	13/0.6	10-30/0.2-1.4
CD16+56+	8/0.4	8-30/0.2-1
**Complete blood cell counts** (At the age of 12)		
Leukocytes (10^3 /µl)	**14.3**	3.7-11.1
Lymphocytes (10^3 /µl)	5.6	1.5-7.6
Neutrophils (10^3 /µl)	**7.7**	1.78-5.38
Platelets (10^3 /µl)	214	140-450
Monocytes (10^3 /µl)	0.7	0.3-0.82
**Lymphocyte subsets** (At the age of 12)	**%/count (10^3 /µl)**	
CD3	**96/5.4**	58-82/1.1-4.1
CD4	**54/3**	26-47/0.6-2.4
CD8	**38/2.1**	16-32/0.4-1.5
CD19	**1/0.06**	10-30/0.2-1.4
CD20	**1/0.06**	11-25/0.2-2
CD16+ CD56+	**2/0.1**	8-30/0.2-1
CD3+ TCRαβ+	90/4.9	78-99 (%)
CD3+ TCRγδ+	5/0.27	1.5-21 (%)
**CD4+ T cell subsets**		
Naive	49/1.4	13-68/0.4-2
Memory	49/1.4	28-68/0.2-1.4
Effector memory	**31/0.9**	3-24 (%)

Central memory	19/0.6	24-51 (%)
Temra	6/0.18	0,3-26 (%)
RTEs	43/1.3	25-63 (%)
CXCR5+ PD1+ (TfH cells)	6.6/0.2	
**CD8+ T cell subsets**		
Naive	78/**1.6**	28-86/0.3-1.5
Memory	21/0.4	12-66/0.05-0.8
Effector memory	15/0.3	6.4–38 (%)
Central memory	4/0.08	1.8–14.2 (%)
Temra	44/0.9	6.2–65.6 (%)
**Immunoglobulins (Before IVIG)**		
IgA (mg/dl)	**10**	62-390
IgG (mg/dl)	**390**	842-1943
IgM (mg/dl)	99	54-392

Bold numbers indicate aberrant values. Temra: Terminally differentiated effector memory
RTE: Recent thymic emigrant TfH: T follicular helper

Supplementary Table 2. Homozygous variants obtained from the filtered exome data

Gene	Chromosome	Position	Reference/Patient	CADD score
IGF2R	6	160469510	C/G	22.9
SIRPB1	20	1592048	G/-	12.7
ZGRF1	4	113539553	T/C	10.6
ANTXRL	10	47701081	C/T	10.7

Supplementary references

1. Halacli SO, Ayvaz DC, Sun-Tan C, Erman B, Uz E, Yilmaz DY, et al. STK4 (MST1) deficiency in two siblings with autoimmune cytopenias: A novel mutation. Clin Immunol. 2015;161(2):316-23.

2. Besci O, Baser D, Ogulur I, Berberoglu AC, Kiykim A, Besci T, et al. Reference values for T and B lymphocyte subpopulations in Turkish children and adults. Turk J Med Sci. 2021;51(4):1814-24.

3. Ikinciogullari A, Kendirli T, Dogu F, Egin Y, Reisli I, Cin S, Babacan E. Peripheral blood lymphocyte subsets in healthy Turkish children. Turk J Pediatr. 2004;46(2):125-30.
